# Acetaminophen and Clinical Outcomes in Sepsis: A Retrospective Propensity Score Analysis of the Ibuprofen in Sepsis Study

**DOI:** 10.1016/j.chstcc.2024.100118

**Published:** 2024-12-10

**Authors:** Sarah N. Obeidalla, Gordon R. Bernard, Lorraine B. Ware, V. Eric Kerchberger

**Affiliations:** Department of Medicine (S. N. O., G. R. B., L. B. W., and V. E. K.), the Department of Pathology, Microbiology and Immunology (L. B. W.), and the Department of Biomedical Informatics (V. E. K.), Vanderbilt University Medical Center, Nashville, TN.

**Keywords:** acetaminophen, mortality, observational study, propensity scores, sepsis/therapy

## Abstract

**BACKGROUND:**

The Ibuprofen in Sepsis Study (ISS) randomized trial found no difference in duration of shock, ARDS, or mortality with ibuprofen treatment for sepsis. However, higher use of acetaminophen, a known hemoprotein reductant with potentially beneficial effects in sepsis, as an antipyretic in the control arm may have masked the clinical benefits from either drug.

**RESEARCH QUESTION:**

Does an association exist between administration of acetaminophen and clinical outcomes in adults with sepsis?

**STUDY DESIGN AND METHODS:**

We performed a retrospective propensity-matched analysis of the previously reported ISS trial. We created a propensity score for receiving acetaminophen during the first 2 study days using sex, age, presence of shock at enrollment, trial study drug assignment (ibuprofen or placebo), febrile status at enrollment, need for mechanical ventilation, and Acute Physiology and Chronic Health Evaluation II score at enrollment, and then matched trial participants 1:1 into acetaminophen-exposed and acetaminophen-unexposed groups based on their propensity scores. We tested the association between receipt of acetaminophen with 30-day mortality as the primary outcome. Secondary outcomes included development of renal failure and ventilator-free days (VFDs).

**RESULTS:**

Of 455 patients in the original trial, 276 patients (61%) were matched into acetaminophen-exposed and acetaminophen-unexposed groups. In the propensity-matched analysis, we found a lower mortality among acetaminophen-exposed patients compared with acetaminophen-unexposed patients (hazard ratio, 0.58; 95% CI, 0.40–0.84; *P* = .004). Additionally, acetaminophen-exposed patients experienced more days alive and free of mechanical ventilation compared with the acetaminophen-unexposed patients (OR, 2.09 for having 19–28 VFDs vs 0 or 1–18 VFDs; 95% CI, 1.12–3.95; *P* = .02). We observed no significant association between renal failure and receipt of acetaminophen.

**INTERPRETATION:**

In this propensity-matched retrospective analysis, adults with sepsis who received acetaminophen showed decreased mortality and more days alive and free of mechanical ventilation. This study highlights the potential of acetaminophen as a modulator of outcomes in sepsis and warrants further investigation.

Sepsis is a nonhomeostatic response to infection leading to severe organ dysfunction with high rates of morbidity and mortality.^1–3^ Current treatment options for sepsis remain limited to antibiotics and supportive care for organ dysfunction,^4^ and a pressing need exists to identify new treatments. Recently, cell-free hemoglobin has been identified as a mediator of oxidative injury and organ dysfunction in sepsis,^5^ and acetaminophen can mitigate this hemoprotein-induced oxidative injury.^6^ Although acetaminophen is used commonly in patients who are critically ill for fever reduction and analgesia, data on the therapeutic effects of acetaminophen in sepsis are conflicting. In an observational cohort of adults with sepsis, patients who received acetaminophen within the first 96 hours of ICU admission showed lower in-hospital mortality.^6^ Another large multicenter observational study of 4 mixed medical-surgical ICUs found that receipt of acetaminophen was associated with lower mortality among a broad swath of critically ill adults.^7^ Furthermore, in a pilot clinical trial in critically ill adults with sepsis with elevated cell-free hemoglobin, patients randomized to 3 days of enteral acetaminophen showed attenuated oxidative stress as measured by plasma F2-isoprostanes and improved serum creatinine compared with patients randomized to placebo.^8^ However, 2 large clinical trials showed no effect of acetaminophen on the primary outcomes compared with placebo. A large randomized multicenter clinical trial of IV acetaminophen in critically ill adults with fever and suspected infection did not observe any differences in ICU-free days or 90-day mortality.^9^ Similarly, a randomized clinical trial of acetaminophen in critically ill adults with sepsis did not find an effect of acetaminophen on 28-day mortality or days alive and free of organ support, although Sequential Organ Failure Assessment scores and the development of ARDS both were reduced significantly with acetaminophen treatment.^10^

Considering this conflicting literature on antipyretics in sepsis, we performed a retrospective analysis of the Ibuprofen in Sepsis Study (ISS) trial,^11^ a randomized clinical trial that compared ibuprofen with placebo in critically ill adults with sepsis. The trial meticulously recorded acetaminophen use for the first 5 days after enrollment. Although the original ISS trial found no effect of ibuprofen on development of ARDS or mortality, higher use of acetaminophen in the placebo arm compared with the intervention arm could have masked the clinical benefits from either drug. Using a propensity-matched analysis of the existing ISS trial data, we evaluated the association between acetaminophen and clinical outcomes, hypothesizing that exposure to acetaminophen was associated with improved outcomes.

## Study Design and Methods

### Study Population and Trial Data Set

The original ISS trial protocol was published previously.^11^ In brief, the trial enrolled critically ill adults at 7 medical centers in the United States and Canada from October 1989 through March 1995 with a known or suspected infection. Inclusion criteria included: a core temperature of at least 38.3 °C or < 35.5 °C, heart rate of ≥ 90 beats/min, and respiratory rate of ≥ 20 breaths/min or ventilatory rate of ≥ 10 L/min if the patient was receiving mechanical ventilation. Patients also had to show evidence of severe cardiovascular, renal, pulmonary, or central nervous system dysfunction. Patients were excluded if they did not meet the inclusion criteria within the first 24 hours of ICU admission and did not receive at least 1 dose of assigned study drug. Other exclusions can be found in the original trial publication.^11^ We included all patients enrolled in the original trial except for patients with missing treatment group identification (ibuprofen or placebo arm), missing clinical data, those who died within 24 hours of trial enrollment, and patients who received their first acetaminophen dose ≥ 48 hours after enrollment. In the ISS trial data set, clinical acetaminophen administration was recorded at baseline, hour 2, then every 4 hours until hour 44, and then once daily until study day 5. Reporting of this study followed the guidelines for Strengthening the Reporting of Observational Studies in Epidemiology.^12^

### Exposure Variable

The exposure variable of interest was receipt of acetaminophen within 2 days of enrollment in the ISS trial.

### Outcome Variables

The primary clinical outcome of interest was 30-day mortality. The secondary clinical outcomes of interest included development of renal failure and ventilator-free days (VFDs) to day 28. All outcome variables were extracted from the original trial data set. VFDs were binned into 3 levels—0 days, 1 to 18 days, and 19 to 28 days—to allow testing by an ordinal logistic regression model while maintaining a clinically meaningful grouping of the outcome.^13^

### Propensity Score Matching

We conducted a propensity-matched analysis to mitigate bias from potential confounders that might be associated both with choice to administer acetaminophen and with clinical outcomes. We generated a propensity score for receiving acetaminophen on the first 2 trial days using a multivariate logistic regression model containing the following variables: sex, age, presence of shock at enrollment, trial study drug assignment (ibuprofen or placebo), febrile status at enrollment, need for mechanical ventilation, and Acute Physiology and Chronic Health Evaluation II score at baseline (e-Fig 1). Using propensity scores, we matched the trial participants in 1:1 pairs of acetaminophen-exposed and acetaminophen-unexposed individuals using nearest neighbor matching with a caliper width of 0.1 SD of the population propensity score to restrict the distance from which patients could be matched. We assessed matching quality by comparing standard mean differences and Kolmogorov-Smirnov statistics for all covariates before and after propensity score matching. We also visually assessed the distributional balance of each matching covariate before and after matching using density plots, bar graphs, and scatterplots as appropriate for each variable. Patients who could not be matched within the specified caliper width were excluded from the propensity-matched analysis.

### Statistical Analyses

We evaluated the association between acetaminophen use and clinical outcomes among the propensity-matched cohort. We used Cox proportional hazard regression, logistic regression, and proportional odds regression for 30-day mortality, renal failure, and VFDs, respectively. Additionally, we specified cluster-robust SEs to account for dependence between observations within matched pairs.^14^ For each outcome, covariates in the multivariable regression models included the same covariates used in the propensity score estimation because using the same covariates can increase precision in the effect estimate and reduce bias resulting from residual imbalance.^15,16^ Subgroup analyses of the model covariates also were performed for all outcomes. We also tested for an interaction effect between acetaminophen and trial-related ibuprofen in separate regression analyses that included an acetaminophen by ibuprofen interaction term. Finally, we used g-computation to estimate the average treatment effect on patients treated with acetaminophen in the propensity-matched cohort. G-computation is a causal inference statistical method that contrasts the potential outcomes among a population at each treatment level, allowing unbiased estimates of the marginal effects of a treatment within a given sample population.^17^ Our g-computation analyses estimated the marginal risk ratio and marginal hazard ratio (HR) for both VFDs and 30-day mortality, respectively.

Sensitivity analyses included (1) stratified propensity matching for acetaminophen use according to ibuprofen treatment arm to account for potential indication bias between acetaminophen use and randomization to the ibuprofen arm (e-Fig 2); (2) recategorizing patients who received acetaminophen only during study days 3 through 5 as nonexposed patients for the purposes of matching, rather than excluding them from matching, to test for potential selection bias in our exclusion criteria; and (3) propensity matching patients who received any acetaminophen across the first 5 study days (e-Fig 3) using the same propensity score methods as the primary analyses. No study size or effect size calculation was performed because this was a reanalysis of previously published trial data. For all analyses, a *P* value of < .05 was considered significant. We used R version 4.2.0 software (R Foundation for Statistical Computing)^18^ for all statistical analyses. R packages used for the matched-cohort analyses included MatchIt,^15^ cobalt,^19^ survival,^20^ RISCA,^21^ and marginaleffects.^22^

## Results

### Patient Characteristics

**Figure 1 –:**
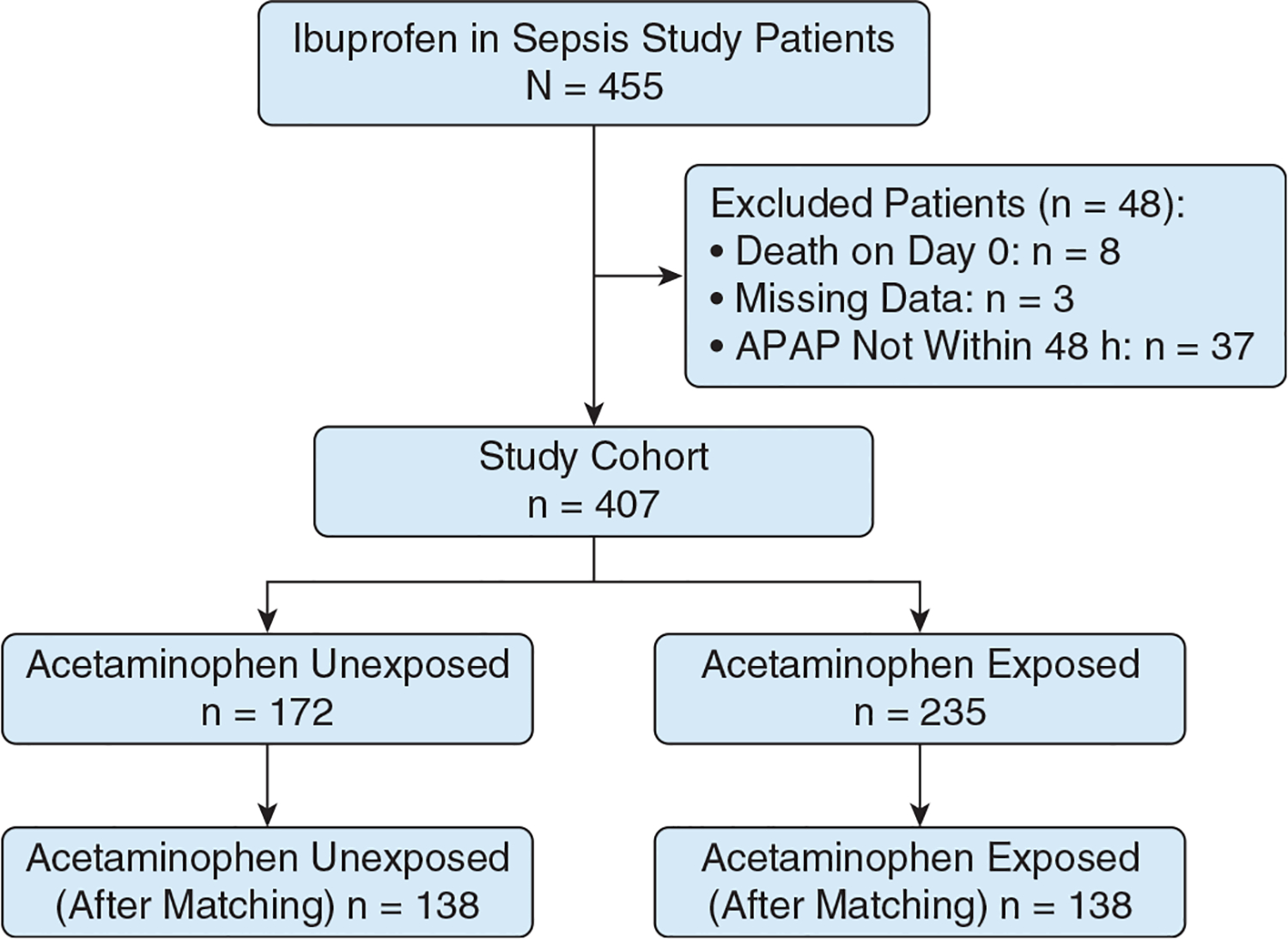
Flowchart showing study cohort. APAP = acetaminophen.

The ISS trial enrolled 455 patients, of which 274 patients (60.0%) received at least 1 dose of acetaminophen during the study. Most acetaminophen-exposed patients (n = 219 [80.0%]) received acetaminophen on the first day of the study (e-Fig 4), with more patients receiving acetaminophen in the placebo trial arm (e-Fig 5). Acetaminophen-exposed patients received a median of 2 doses (interquartile range, 1–3 doses) during the first 48 hours of the study. We excluded 3 patients because of missing data, 8 patients because of death within 24 hours of enrollment, and 37 patients for receiving the first dose of acetaminophen after the 48-hour mark (Fig 1). After propensity matching, we matched 276 patients (68.0%) into 138 acetaminophen-exposed and unexposed pairs. Table 1 shows the cohort characteristics both before and after matching, along with the covariates and outcomes used in the models. Additional patient characteristic tables for our supplementary analyses can be found in e-Tables 1 and 2. We achieved satisfactory matching for all covariates based on visual inspection of distributional plots, as well as postmatching standard mean differences and Kolmogorov-Smirnov statistics of < 0.1 for all matching variables (e-Figs 6, 7).

### Propensity-Matched Analysis

**Figure 2 –:**
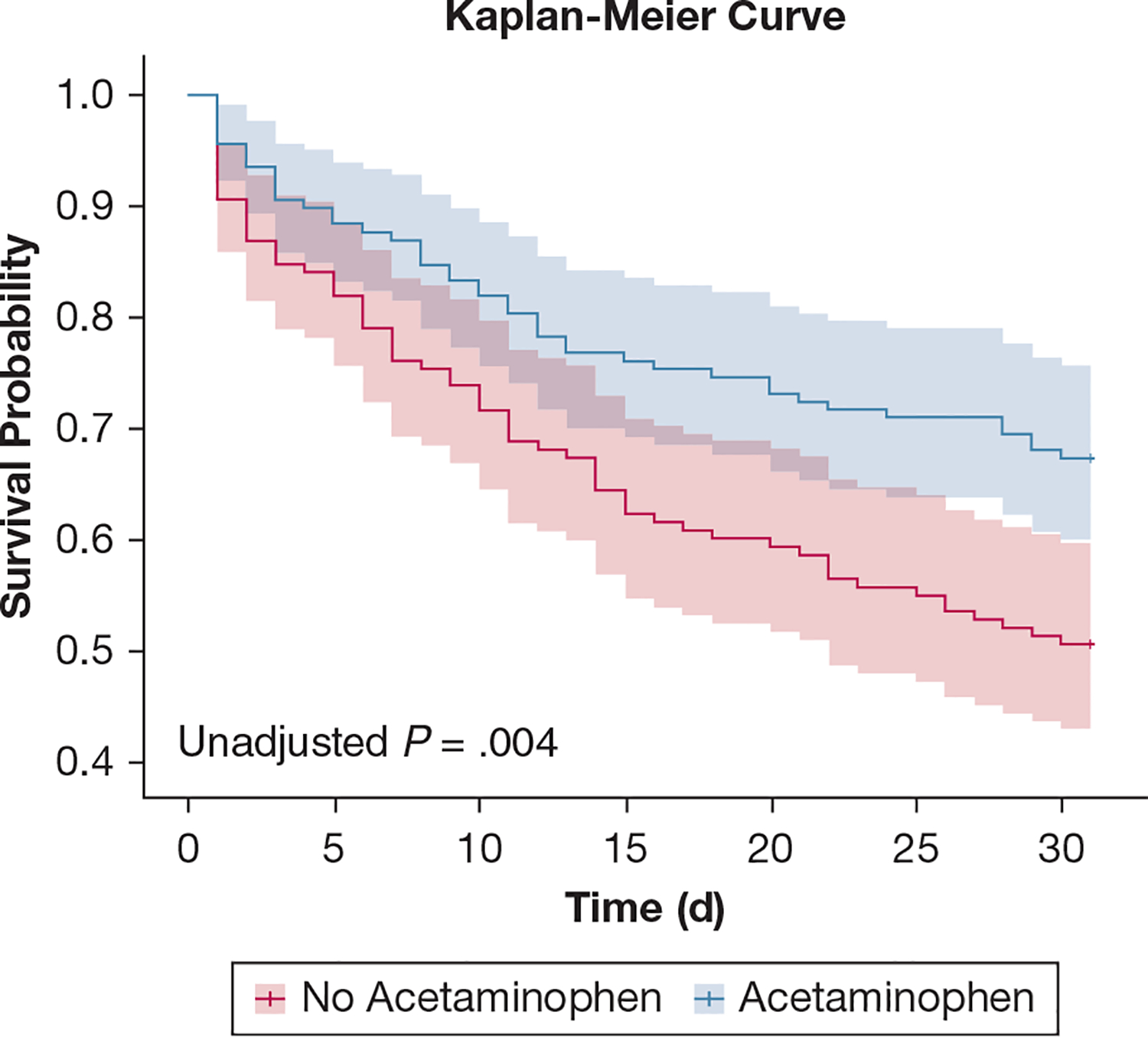
Kaplan-Meier survival curves for the propensity matched analysis. Survival probability over time in the propensity-matched cohort up to 30 days after enrollment in the trial.

**Figure 3 –:**
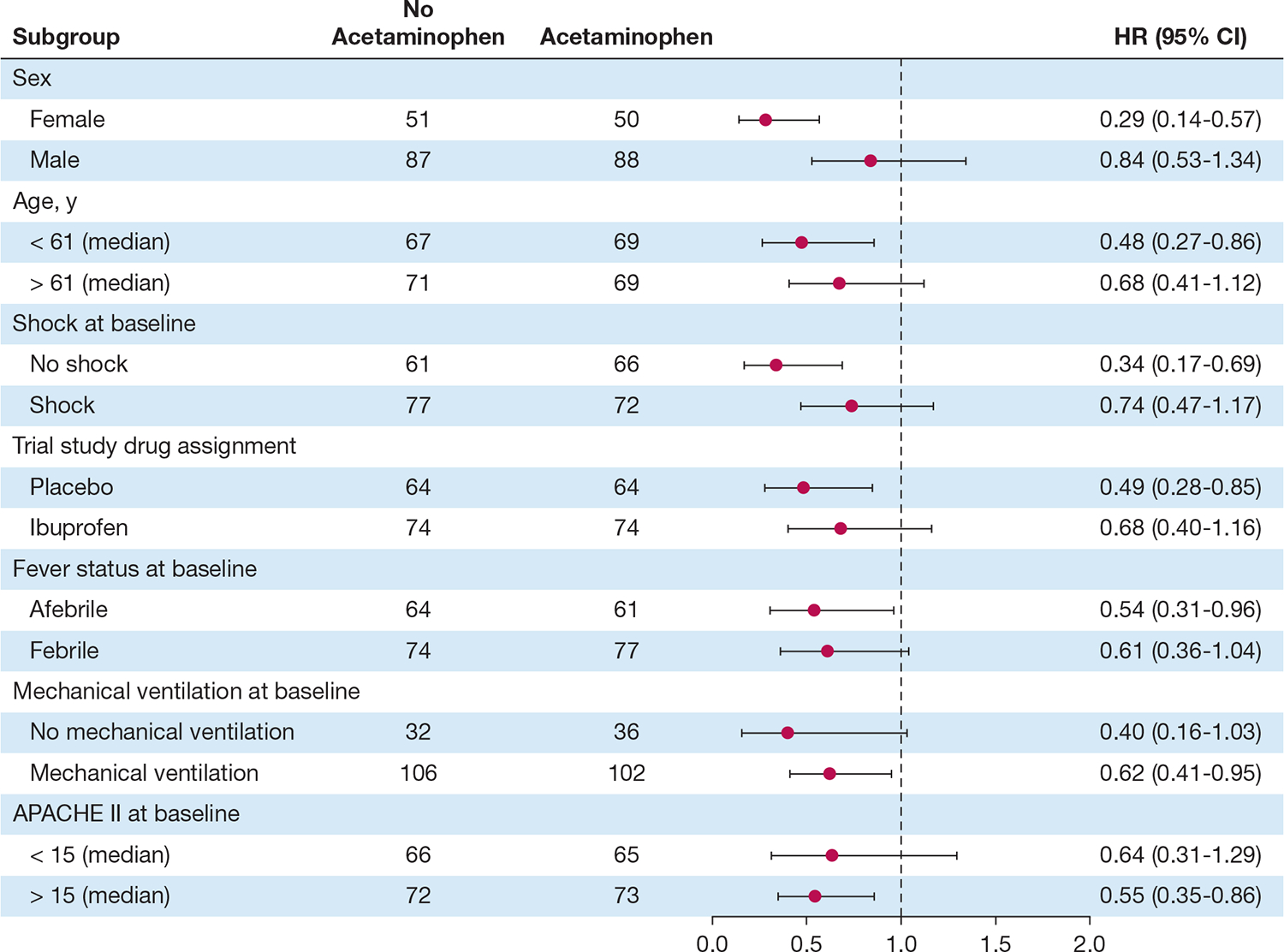
Subgroup analysis of acetaminophen effect on survival across covariate subgroups in the propensity matched cohort. The dots are point estimates, the bars are CIs, and the vertical dotted line represents a hazard ratio (HR) of 1. APACHE = Acute Physiology and Chronic Health Evaluation.

**Figure 4 –:**
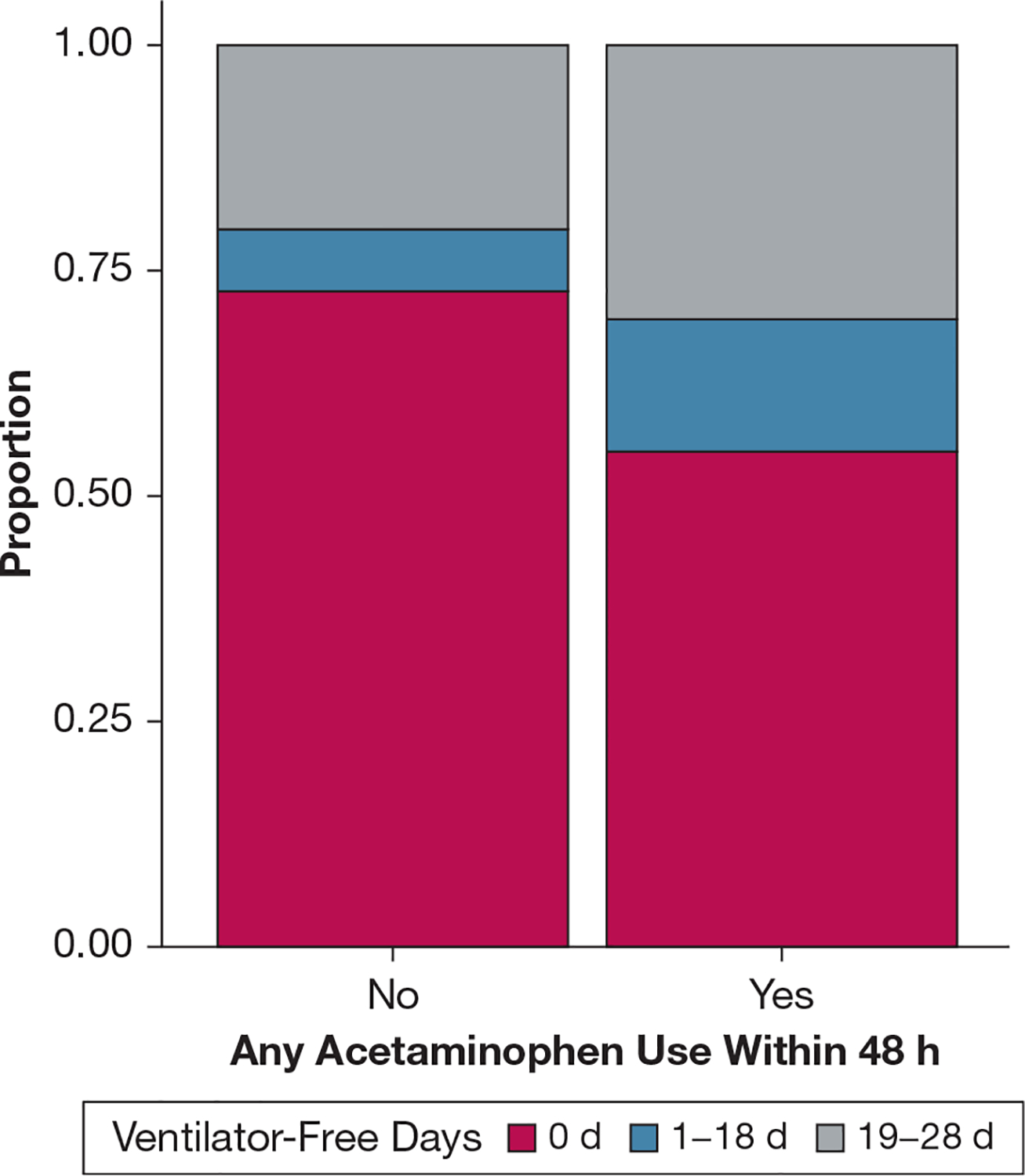
Comparison of ventilator-free days between acetaminophen-exposed and acetaminophen-unexposed patients in the propensity matched analysis.

In our primary propensity-matched analysis, we observed a significantly lower mortality risk at 30 days among acetaminophen-exposed patients compared with acetaminophen-unexposed patients (HR, 0.58; 95% CI, 0.40–0.84; *P* = .004) (Fig 2). G-computation of the marginal HR of acetaminophen-exposed patients showed a 37% lower risk of death at 30 days (HR, 0.63; 95% CI, 0.48–0.81; *P* < .001). The risk of death was consistently lower with acetaminophen across all clinical subgroups in the propensity-matched analysis (Fig 3). Similarly, acetaminophen-exposed patients experienced more days alive and free of mechanical ventilation compared with acetaminophen-unexposed patients (OR, 2.09 for having 19–28 VFDs vs 0 VFDs or 1–18 VFDs; 95% CI, 1.12–3.95; *P* = .02) (Fig 4). G-computation of the marginal risk ratio (RR) for being in each VFD level showed that acetaminophen-exposed patients had a 22% lower probability of being in the 0 VFD group (RR, 0.78; 95% CI, 0.63–0.96; *P* = .02), a 22% higher probability of being in the 1 to 18 VFDs group (RR, 1.22; 95% CI, 1.00–1.48; *P* = .05), and a 62% higher probability of being in the 19 to 28 VFDs group (RR, 1.62; 95% CI, 1.08–2.43; *P* = .02). This effect was consistent across all subgroups of the clinical covariates (e-Fig 8). In contrast, we observed no difference in development of renal failure between acetaminophen-exposed and acetaminophen-unexposed patients (OR, 1.5; 95% CI, 0.66–3.4; *P* = .35) (e-Fig 9). In the interaction analyses, we did not observe any evidence of effect modification between acetaminophen and trial-related ibuprofen on mortality (HR for interaction term, 1.40; 95% CI, 0.65–3.05; *P* = .4) (e-Table 3), VFDs (OR for interaction term, 0.64; 95% CI, 0.18–2.25; *P* = .49), or renal failure (OR for interaction term, 0.61; 95% CI, 0.10–3.43; *P* = .57).

### Sensitivity Analyses

When the propensity matching was stratified by ibuprofen treatment arm, we observed similarly decreased mortality risk at 30 days and similarly increased VFDs among acetaminophen-exposed patients (e-Fig 10). Furthermore, when we recategorizing patients whose acetaminophen-exposure occurred only late in the study (days 3–5) as nonexposed rather than as excluded, we observed similar results showing decreased mortality risk at 30 days and increased VFDs among acetaminophen-exposed patients (e-Table 4). We also observed similar results when analyzing receipt of acetaminophen across the first 5 days of the study (e-Fig 11).

## Discussion

### Primary Findings

We performed a retrospective analysis of a previously published clinical trial to examine the association between acetaminophen and clinical outcomes among critically ill adults with sepsis. The ISS was chosen for this analysis because it included meticulous collection of acetaminophen administration for the first 5 days of enrollment and because fever reduction in the ibuprofen arm caused the group randomized to the placebo arm to have higher acetaminophen use. Given the potential beneficial effect of acetaminophen as a hemoprotein reductant in sepsis, we hypothesized that open-label acetaminophen may have impacted the original trial results. Although we found no evidence of effect modification between open-label use of acetaminophen and trial-related ibuprofen, our propensity-matched analyses demonstrated that acetaminophen-exposed patients showed a lower 30-day mortality risk and had more days alive and free of mechanical ventilation compared with acetaminophen-unexposed patients, independent of trial-related ibuprofen assignment. In contrast, no difference was found in development of renal failure between acetaminophen-exposed and unexposed patients. In further support of the robustness of these findings, results were similar when examining acetaminophen use across all 5 study days and when stratifying the propensity matching by ibuprofen treatment arm.

### Relationship to Prior Literature

Acetaminophen may exert protective effects in patients who are critically ill by reducing cell-free hemoglobin-induced oxidative injury.^6,8^ Our study adds to the literature supporting a beneficial effect of acetaminophen in patients with sepsis and other clinical conditions associated with high oxidative stress. In a large multicenter observational study of 15,818 critically ill adults, acetaminophen use was associated with lower mortality across multiple subgroup analyses.^7^ In contrast, acetaminophen did not lead to more ICU-free days compared with placebo in a clinical trial of 700 patients who were critically ill with suspected infection.^9^ However, high rates of open-label acetaminophen use in both trial arms could have masked potential benefits, and a secondary analysis of that trial did identify heterogeneity in response to acetaminophen, with acetaminophen being associated with shorter ICU length of stay among survivors, but a longer ICU length of stay among nonsurvivors.^9^ More recently, a clinical trial of acetaminophen in patients with sepsis and respiratory or circulatory organ dysfunction also did not identify a difference in mortality with acetaminophen.^10^ However, in prespecified secondary analyses, patients in the acetaminophen arm experienced lower rates of ARDS and greater improvement in Sequential Organ Failure Assessment scores.^10^ Thus, although the current study supports a benefit of acetaminophen in severe sepsis, larger prospective studies are still needed.

Our study did not recapitulate previous observations of acetaminophen exerting a protective effect on renal function, including lower rate of acute kidney injury among children undergoing cardiac surgery,^23^ improved serum creatinine level among adults with severe falciparum malaria,^24^ and improved serum creatinine level among adults with sepsis and elevated cell-free hemoglobin level.^8^ However, the ISS trial data set captured only the presence or absence of renal failure as defined in the original trial by urine output of < 30 mL/h or < 0.5 mL/h/kg body weight for at least 1 hour. Daily creatinine values were available in the trial data set; however, no substantial differences were found between acetaminophen use and daily creatinine (not shown).

### Strengths and Limitations

This study has several strengths. We used data from a well-designed randomized controlled clinical trial in sepsis and had detailed data on acetaminophen administration during the trial. Our propensity score matching resulted in excellent balance between the acetaminophen-exposed and unexposed groups. Propensity-matched analyses are a useful tool for causal inference and have been applied widely in critical care research. Previous examples include studies observing a lower risk of ARDS among adults with prehospital aspirin use^25^ and a study that found reduced in-hospital mortality in patients prescribed paracetamol in the ICU.^7^ Our findings also were robust to several sensitivity analyses, and our use of g-computation allows for consistent estimates of the average treatment effect among patients exposed to acetaminophen.^26^

Our study also has some limitations. Although patients in the original ISS trial were randomized to ibuprofen vs placebo, the use of acetaminophen in the trial was not randomized and therefore is at risk of confounding by indication. Although the propensity score matching mitigates this concern by balancing covariate distributions between the exposed and unexposed groups, a risk of residual bias resulting from unmeasured confounders remains.^27^ Our study also could not differentiate administration of acetaminophen for fever, analgesia, or both. However, fever was very common in patients both exposed and unexposed to acetaminophen, suggesting that the risk of confounding by indication may not be substantial. Additionally, the ISS trial was conducted several decades ago. Although all common interventions currently used for sepsis management (antibiotics and source control of infection, IV fluids, and vasoactive agents for hypotension) were available and were used commonly when the ISS trial was published,^28^ overall sepsis mortality has declined substantially over time, although to date no clinical therapeutic drug has demonstrated a consistent effect on sepsis mortality.^29,30^ These improvements likely reflect increased sepsis recognition,^31^ emphasis on timely sepsis identification and initiation of antibiotics,^32^ advances in supportive care such as lung-protective ventilation in ARDS, and daily spontaneous awakening and breathing trials in patients who were ventilated.^33,34^ However, despite modern supportive care measures, sepsis-related morbidity and mortality remain quite high, which emphasizes the need for further prospective studies of potential therapeutics such as acetaminophen in sepsis. Plasma cell-free hemoglobin levels were not available from the ISS trial data set. Because the putative beneficial effect of acetaminophen in sepsis is the result of hemoprotein reductant activity, more benefit might be apparent in patients with elevated cell-free hemoglobin.^6^ Additionally, propensity matching did reduce the sample size in our matched analyses, potentially reducing study power to detect smaller differences in other outcomes, such as acute renal failure. Finally, although we observed an improvement in clinical outcomes with receipt of acetaminophen, it is possible that unmeasured confounders remain.

## Interpretation

In a propensity-matched reanalysis of the ISS, patients who were critically ill who received acetaminophen showed higher survival rates and more days free of mechanical ventilation, but no difference in development of renal failure, compared with patients who did not receive acetaminophen. Although this retrospective study carries some limitations, our findings provide support for a potential beneficial effect of acetaminophen in sepsis and underscore the need for additional prospective clinical trials to identify subgroups of patients who may benefit from acetaminophen.

## Supplementary Material

1

## Figures and Tables

**TABLE 1]:** Cohort Characteristics Both Before and After Matching, Along With Covariates and Outcomes Used in Models

Characteristic	Unmatched		Matched	
	No Acetaminophen (n = 172)	Acetaminophen (n = 235)	No Acetaminophen (n = 138)	Acetaminophen (n = 138)
Sex				
Female	68 (40)	89 (38)	51 (37)	50 (36)
Male	104 (60)	146 (62)	87 (63)	88 (64)
White race	112 (65)	151 (64)	93 (67)	97 (70)
Age	63 (52–71)	52 (37–67)	61 (47–69)	61 (47–69)
Shock at baseline	99 (58)	109 (46)	77 (56)	72 (52)
Randomized to ibuprofen	96 (56)	100 (43)	74 (54)	74 (54)
Febrile status at baseline				
Afebrile	92 (53)	68 (29)	64 (46)	61 (44)
Febrile	80 (47)	167 (71)	74 (54)	77 (56)
Mechanical ventilation at baseline	132 (77)	180 (77)	106 (77)	102 (74)
Ventilator-free days				
0	94 (73)	75 (48)	74 (73)	47 (55)
1–18	9 (7.0)	20 (13)	7 (6.9)	13 (15)
19–28	26 (20)	61 (39)	21 (21)	26 (30)
APACHE II score at baseline	16 (11–22)	14 (9–19)	15 (10–20)	15 (11–21)
Death at 30 d	87 (51)	70 (30)	67 (49)	44 (32)
APAP dose at 48 h	0 (0–0)	2 (1–3)	0 (0–0)	1 (1–3)

Data are presented as No. (%) or median (interquartile range). APACHE = Acute Physiology and Chronic Health Evaluation.

## Take-Home Points

### Study Question:

Is administration of acetaminophen associated with improved clinical outcomes in adults with sepsis?

### Results:

In a propensity-matched retrospective analysis of a previously published clinical trial, patients with sepsis who received acetaminophen showed higher survival rates and more days free of mechanical ventilation, but no difference in development of renal failure, compared with patients who did not receive acetaminophen.

### Interpretation:

Our findings provide support for a potential beneficial effect of acetaminophen in sepsis.

## ABBREVIATIONS:

HR: hazard ratio
ISS: Ibuprofen in Sepsis Study
VFD: ventilator-free day
