# Supplementary material for: Acetaminophen and Clinical Outcomes in Sepsis: A Retrospective Propensity Score Analysis of the Ibuprofen in Sepsis Study
Source: CHEST Crit Care. Author manuscript; Available in PMC 2025 Mar 25. (PMC11936508; doi:10.1016/j.chstcc.2024.100118)

**Acetaminophen and Clinical Outcomes in Sepsis: A Retrospective Propensity Score Analysis of the Ibuprofen in Sepsis Study.**

**Supplemental Materials**

Table of Contents

[Table of Contents 1](#_Toc181621570)

[e-Table 1. Patient Characteristics by Acetaminophen use During First Two Study Days in a Propensity Matched Cohort Stratified by Treatment Arm 2](#_Toc181621571)

[e-Table 2. Patient Characteristics by Acetaminophen use During First Five Study Days in a Propensity Matched Cohort 3](#_Toc181621572)

[e-Table 3. Interaction Model Results between Acetaminophen and Ibuprofen Use in the Propensity Matched Cohort. 4](#_Toc181621573)

[e-Table 4. Sensitivity Analysis: Re-categorizing patients with late acetaminophen exposure (only on study days 3-5) as “non-exposed” prior to matching. 5](#_Toc181621574)

[e-Figure 1. Directed Acyclic Graph for Propensity Score 6](#_Toc181621575)

[e-Figure 2. Study Cohort Flowchart for Acetaminophen use within the first two days in a Stratified Propensity Matched Analysis by Treatment Arm 7](#_Toc181621576)

[e-Figure 3. Study Cohort Flowchart for Acetaminophen use within the First Five Days 8](#_Toc181621577)

[e-Figure 4. Acetaminophen Use During First Five Study Days 9](#_Toc181621578)

[e-Figure 5. Acetaminophen Use During First Five Study Days by Treatment Arm in the ISS trial. 10](#_Toc181621579)

[e-Figure 6. Love Plot for distributional balance of before and after propensity score matching. 11](#_Toc181621580)

[e-Figure 7. Distributional balance of covariates before and after propensity-score matching. 12](#_Toc181621581)

[e-Figure 8. Subgroup analyses of acetaminophen effect on ventilator free days across covariate subgroups in the Propensity Matched Cohort 13](#_Toc181621582)

[e-Figure 9. Subgroup analyses of acetaminophen effect on renal failure across covariate subgroups in the Propensity Matched Cohort 14](#_Toc181621583)

[e-Figure 10. (A) Kaplan-Meier Survival Curve for Stratified Propensity Matched Analysis of Acetaminophen use within the First Two Days and (B) Comparison of Ventilator-Free Days between Acetaminophen-Exposed and Acetaminophen-Unexposed Patients in the Stratified Propensity Matched Analysis in the First Two Days. 15](#_Toc181621584)

[e-Figure 11. (A) Kaplan-Meier Survival Curve for Propensity Matched Analysis of Acetaminophen use within the First Five Days and (B) Comparison of Ventilator-Free Days between Acetaminophen-Exposed and Acetaminophen-Unexposed Patients in the Propensity Matched Analysis in the First Five Days. 16](#_Toc181621585)

# e-Table 1. Patient Characteristics by Acetaminophen use During First Two Study Days in a Propensity Matched Cohort Stratified by Treatment Arm

| **Characteristic** | **No Acetaminophen**, N = 127*^1^* | **Acetaminophen**, N = 127*^1^* |
| --- | --- | --- |
| Sex |  |  |
| Female | 47 (37%) | 49 (39%) |
| Male | 80 (63%) | 78 (61%) |
| White Race | 83 (65%) | 82 (65%) |
| Age | 61 (44, 69) | 60 (41, 70) |
| Shock at Baseline | 69 (54%) | 70 (55%) |
| Randomized to Ibuprofen | 70 (55%) | 70 (55%) |
| Febrile Status at Baseline |  |  |
| Afebrile | 57 (45%) | 54 (43%) |
| Febrile | 70 (55%) | 73 (57%) |
| Mechanical Ventilation at Baseline | 94 (74%) | 91 (72%) |
| Ventilator Free Days |  |  |
| 0 Days | 70 (75%) | 40 (51%) |
| 1-18 Days | 5 (5.4%) | 10 (13%) |
| 19-28 Days | 18 (19%) | 29 (37%) |
| APACHE II Score at Baseline | 15 (10, 20) | 14 (10, 21) |
| Death at 30 Days | 64 (50%) | 37 (29%) |
| *^1^*n (%); Median (IQR) | | |

# e-Table 2. Patient Characteristics by Acetaminophen use During First Five Study Days in a Propensity Matched Cohort

| **Characteristic** | **No Acetaminophen**, N = 152*^1^* | **Acetaminophen**, N = 152*^1^* |
| --- | --- | --- |
| Sex |  |  |
| Female | 60 (39%) | 52 (34%) |
| Male | 92 (61%) | 100 (66%) |
| White Race | 104 (68%) | 100 (66%) |
| Age | 62 (50, 69) | 61 (47, 70) |
| Shock at Baseline | 87 (57%) | 78 (51%) |
| Randomized to Ibuprofen | 82 (54%) | 81 (53%) |
| Febrile Status at Baseline |  |  |
| Afebrile | 73 (48%) | 74 (49%) |
| Febrile | 79 (52%) | 78 (51%) |
| Mechanical Ventilation at Baseline | 117 (77%) | 110 (72%) |
| Ventilator Free Days |  |  |
| 0 Days | 81 (71%) | 53 (51%) |
| 1-18 Days | 9 (7.9%) | 11 (11%) |
| 19-28 Days | 24 (21%) | 40 (38%) |
| APACHE II Score at Baseline | 15 (11, 20) | 14 (10, 19) |
| Death at 30 Days | 75 (49%) | 52 (34%) |
| *^1^*n (%); Median (IQR) | | |

# e-Table 3. Interaction Model Results between Acetaminophen and Ibuprofen Use in the Propensity Matched Cohort.

|  | **Mortality at 30 Days** | | | **Ventilator Free Days** | | | **Renal Failure** | | |
| --- | --- | --- | --- | --- | --- | --- | --- | --- | --- |
| *Predictors* | *Estimates* | *CI* | *p* | *Odds Ratios* | *CI* | *p* | *Odds Ratios* | *CI* | *p* |
| Acetaminophen use within 2 Days | 0.49 | 0.28 – 0.85 | **0.017** | 2.68 | 1.06 – 7.13 | **0.043** | 2.06 | 0.51 – 8.77 | 0.315 |
| Randomized to Ibuprofen | 0.61 | 0.38 – 1.00 | 0.053 | 2.44 | 0.96 – 6.53 | 0.067 | 2.10 | 0.65 – 7.22 | 0.221 |
| Male | 1.01 | 0.69 – 1.50 | 0.944 | 1.14 | 0.59 – 2.24 | 0.699 | 0.77 | 0.31 – 1.87 | 0.567 |
| Age | 0.99 | 0.98 – 1.01 | 0.459 | 1.00 | 0.98 – 1.03 | 0.682 | 1.02 | 0.99 – 1.05 | 0.171 |
| Shock at Baseline | 1.90 | 1.25 – 2.87 | **0.002** | 0.74 | 0.38 – 1.46 | 0.388 | 1.04 | 0.44 – 2.43 | 0.928 |
| Fever | 0.89 | 0.60 – 1.31 | 0.552 | 0.99 | 0.52 – 1.91 | 0.986 | 1.26 | 0.52 – 3.03 | 0.603 |
| Mechanical Ventilation at Baseline | 1.27 | 0.75 – 2.15 | 0.378 |  |  |  | 3.45 | 1.35 – 9.41 | **0.012** |
| APACHE II at Baseline | 1.08 | 1.05 – 1.10 | **<0.001** | 0.89 | 0.84 – 0.94 | **<0.001** | 0.99 | 0.93 – 1.05 | 0.694 |
| Acetaminophen x Ibuprofen | 1.40 | 0.65 – 3.05 | 0.400 | 0.64 | 0.18 – 2.25 | 0.485 | 0.61 | 0.10 – 3.43 | 0.571 |
| 0 Days\|1-18 Days |  |  |  | 0.83 | 0.19 – 3.59 | 0.807 |  |  |  |
| 1-18 Days\|19-28 Days |  |  |  | 1.50 | 0.35 – 6.46 | 0.585 |  |  |  |
| Observations | 276 | | | 188 | | | 108 | | |
| R^2^ Nagelkerke | 0.193 | | | 0.181 | | | 0.122 | | |

# e-Table 4. Sensitivity Analysis: Re-categorizing patients with late acetaminophen exposure (only on study days 3-5) as “non-exposed” prior to matching.

|  | **Mortality at 30 Days** | | | **Ventilator Free Days** | | | **Renal Failure** | | |
| --- | --- | --- | --- | --- | --- | --- | --- | --- | --- |
| *Predictors* | *Estimates* | *CI* | *p* | *Odds Ratios* | *CI* | *p* | *Odds Ratios* | *CI* | *p* |
| Acetaminophen use within 2 Days | 0.63 | 0.44 – 0.91 | **0.012** | 1.92 | 1.09 – 3.42 | **0.025** | 1.01 | 0.47 – 2.17 | 0.987 |
| Male | 1.15 | 0.79 – 1.68 | 0.502 | 1.07 | 0.60 – 1.91 | 0.831 | 0.56 | 0.25 – 1.24 | 0.157 |
| Age | 1.00 | 0.99 – 1.01 | 0.851 | 1.01 | 0.99 – 1.03 | 0.529 | 1.03 | 1.00 – 1.05 | **0.033** |
| Shock at Baseline | 1.71 | 1.15 – 2.55 | **0.006** | 0.69 | 0.39 – 1.24 | 0.219 | 1.26 | 0.56 – 2.81 | 0.572 |
| Randomized to Ibuprofen | 0.78 | 0.54 – 1.12 | 0.144 | 1.43 | 0.81 – 2.55 | 0.226 | 1.83 | 0.84 – 4.08 | 0.131 |
| Fever | 0.73 | 0.50 – 1.07 | 0.115 | 1.50 | 0.82 – 2.75 | 0.191 | 1.65 | 0.73 – 3.77 | 0.226 |
| Mechanical Ventilation at Baseline | 1.19 | 0.73 – 1.95 | 0.512 |  |  |  | 1.72 | 0.69 – 4.40 | 0.247 |
| APACHE II at Baseline | 1.08 | 1.05 – 1.11 | **<0.001** | 0.88 | 0.84 – 0.92 | **<0.001** | 1.00 | 0.95 – 1.07 | 0.869 |
| 0 Days\|1-18 Days |  |  |  | 0.48 | 0.13 – 1.80 | 0.275 |  |  |  |
| 1-18 Days\|19-28 Days |  |  |  | 0.81 | 0.21 – 3.03 | 0.748 |  |  |  |
| Observations | 314 | | | 219 | | | 121 | | |
| R^2^ Nagelkerke | 0.178 | | | 0.217 | | | 0.111 | | |

# e-Figure 1. Directed Acyclic Graph for Propensity Score


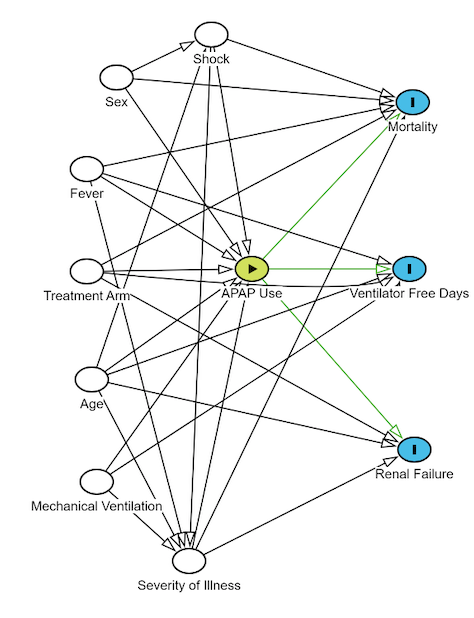


The graph shows the relationship among the outcomes (30-day mortality, ventilator-free days, and renal failure), the exposure (acetaminophen use within 48 hours), and the covariates (sex, age, treatment arm, shock at enrollment, fever status at enrollment, need for mechanical ventilation, and APACHE II score). Arrows depict direction of effect between covariates with the exposure and/or the outcome(s).

# e-Figure 2. Study Cohort Flowchart for Acetaminophen use within the first two days in a Stratified Propensity Matched Analysis by Treatment Arm


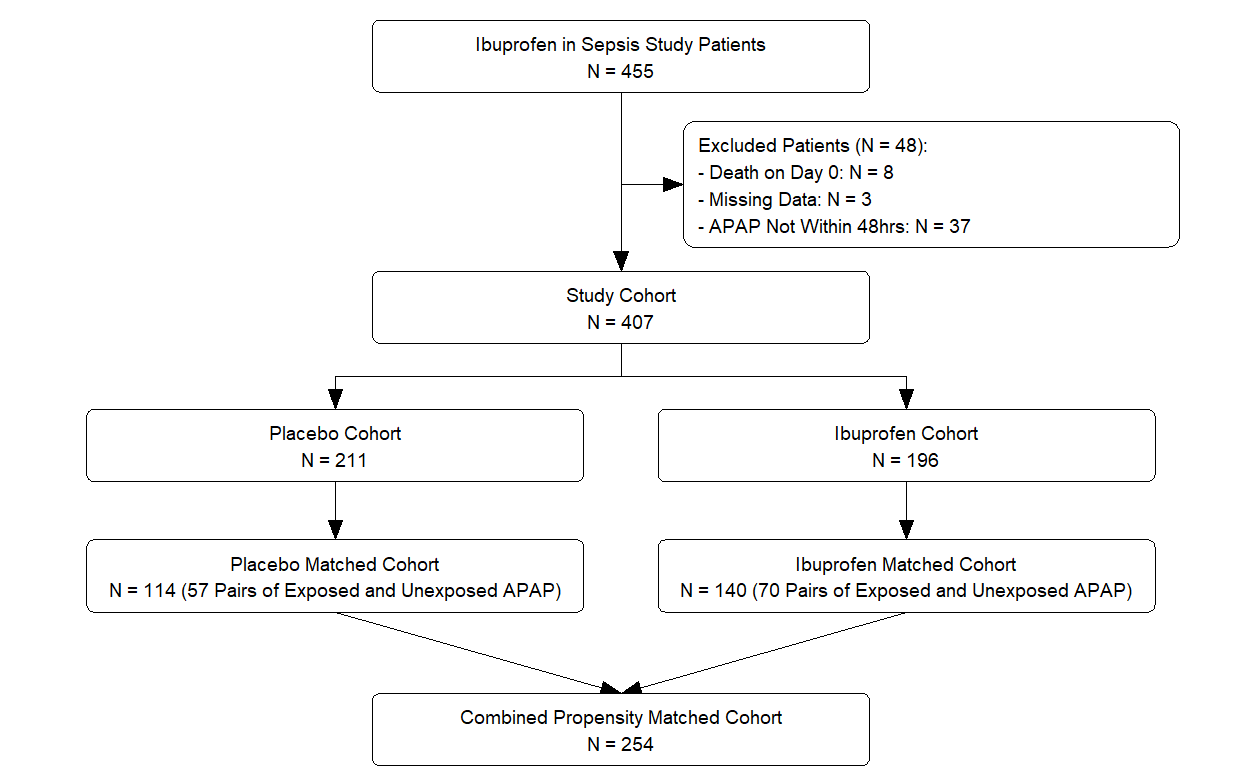


# e-Figure 3. Study Cohort Flowchart for Acetaminophen use within the First Five Days


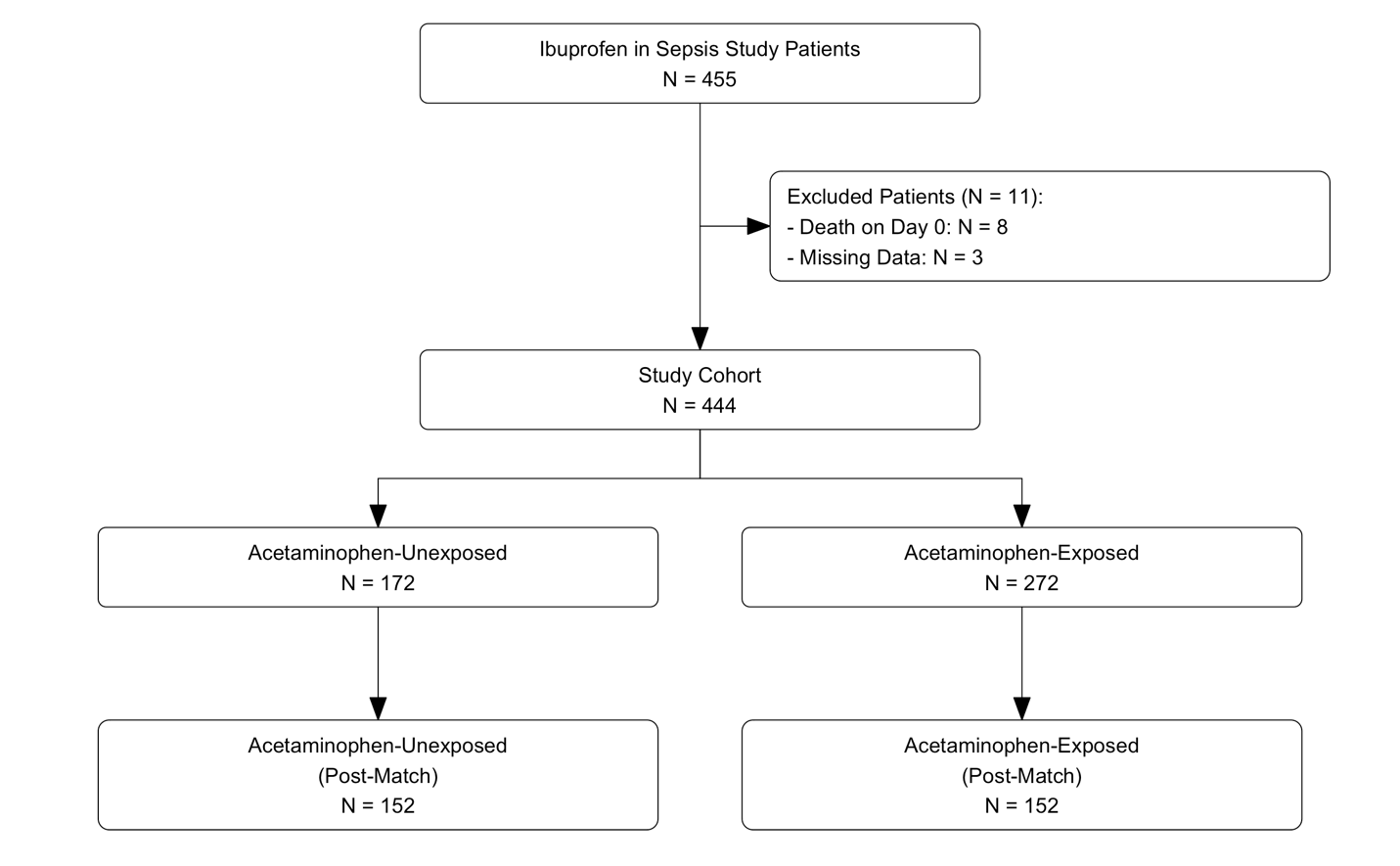


# e-Figure 4. Acetaminophen Use During First Five Study Days


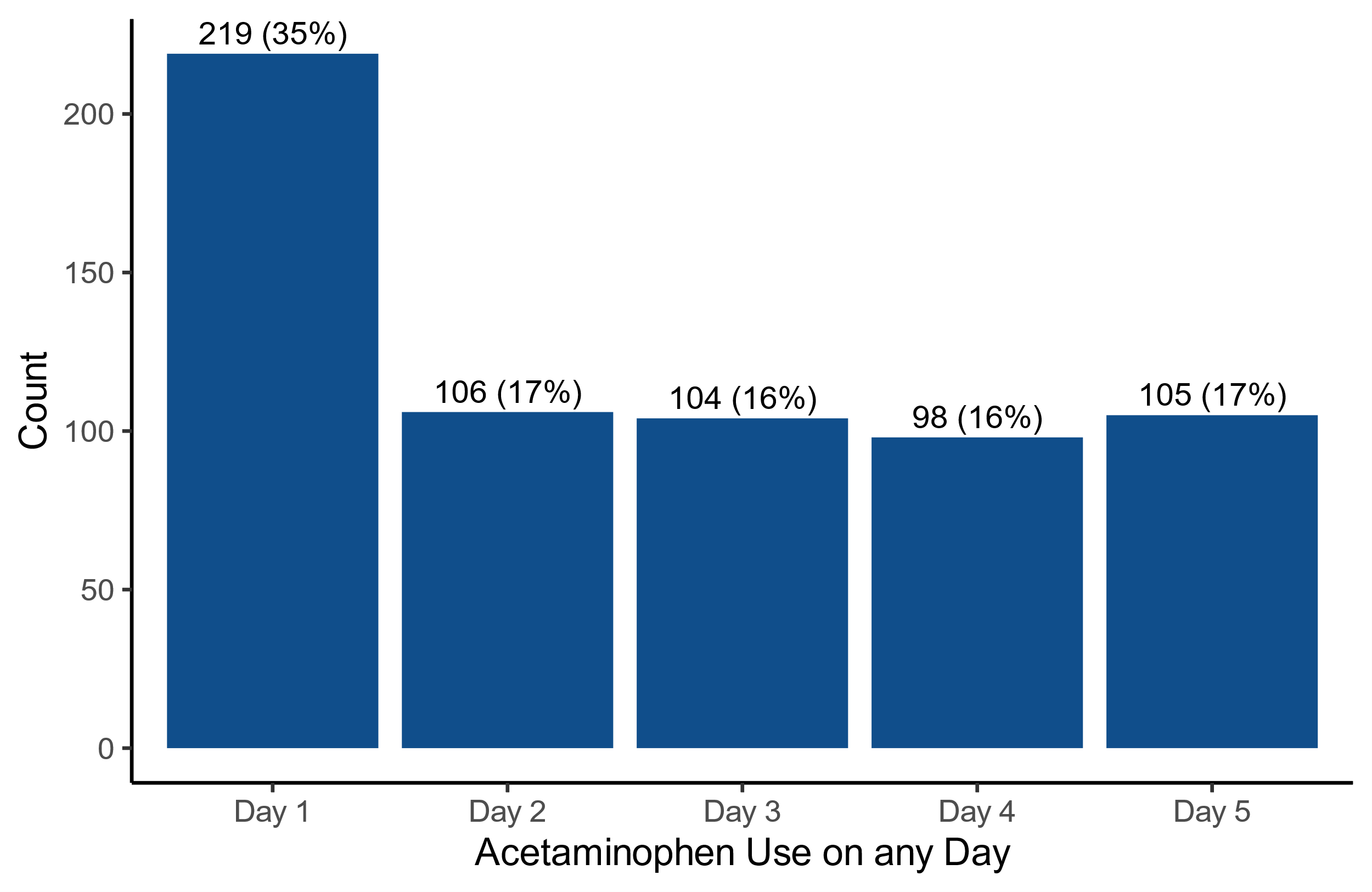


Labels show number (%) of patients receiving acetaminophen during each of the first five study days in the overall Ibuprofen in Sepsis Study (ISS) cohort.

# e-Figure 5. Acetaminophen Use During First Five Study Days by Treatment Arm in the ISS trial.


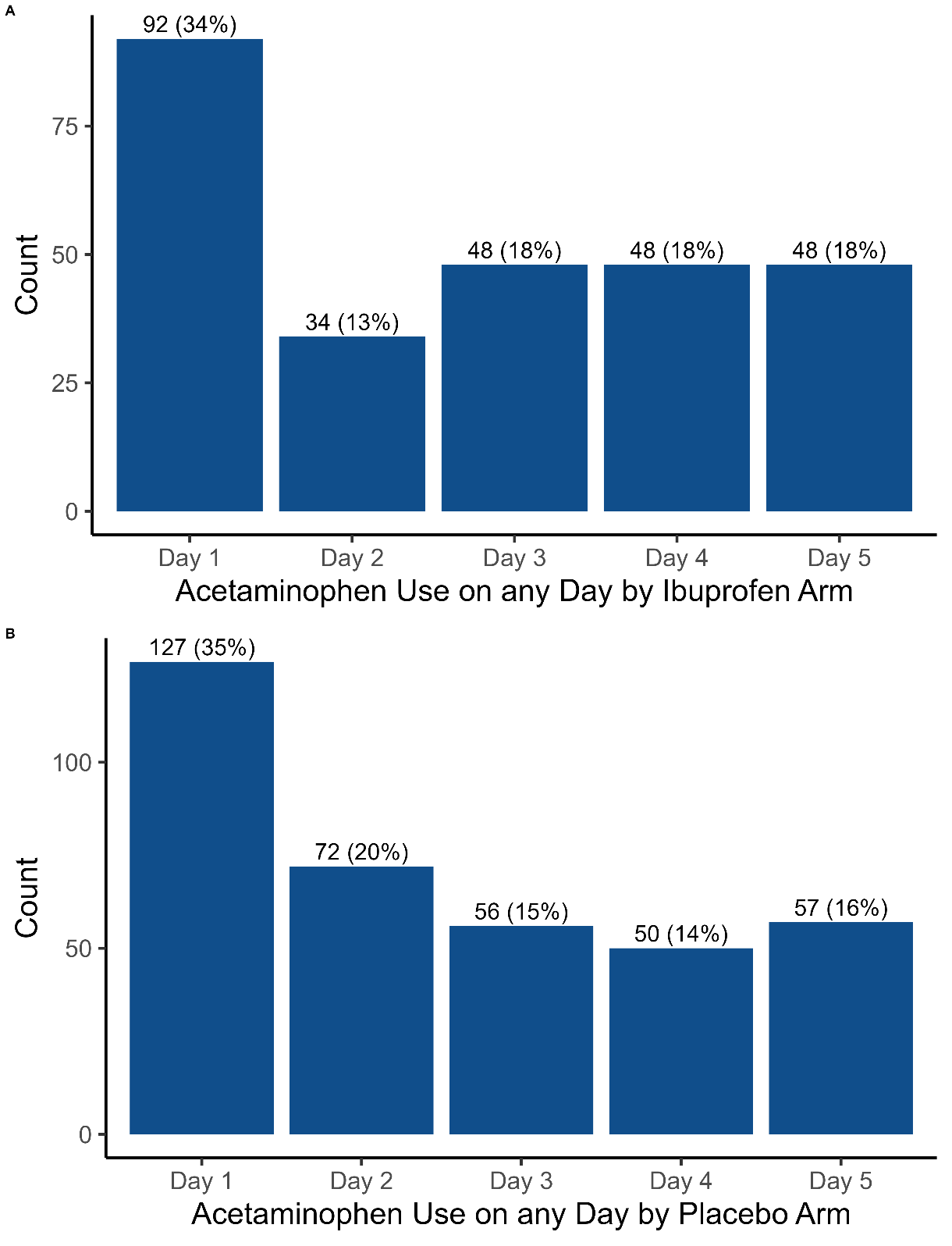


Labels show number (%) of patients receiving acetaminophen during each of the first five study days for the (A) Ibuprofen arm and (B) placebo arm.

# e-Figure 6. Love Plot for distributional balance of before and after propensity score matching.


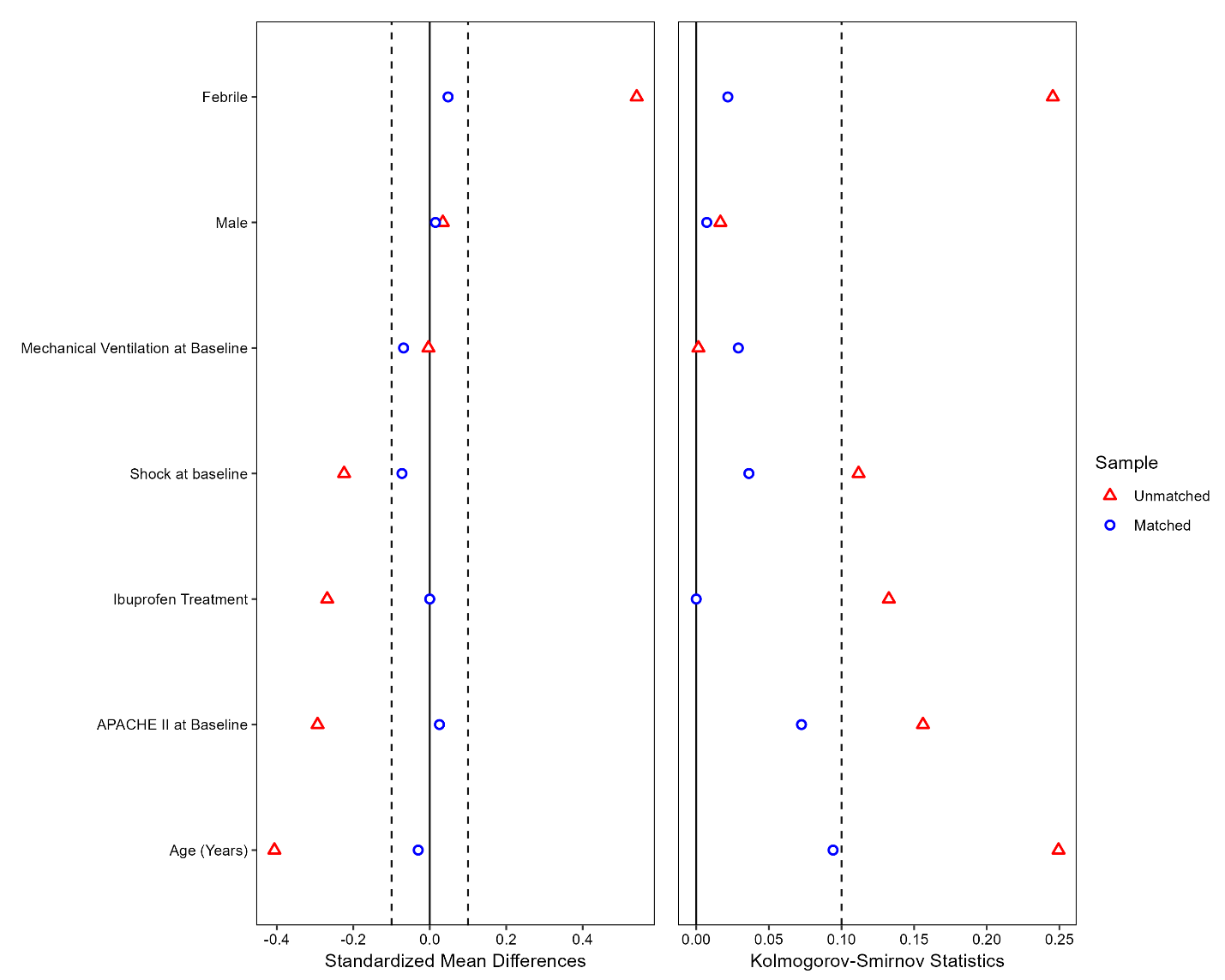


Love Plot of differences in matching variables between acetaminophen-exposed and acetaminophen-unexposed patients. Left plot shows standardized mean differences for each variable, right plot shows the Kolmogorov-Smirnov statistic for each variable. Red triangles show values for each variable in the full unmatched cohort, blue circles show values for each variable after matching. Dotted vertical lines show thresholds for acceptable matching for each variable.

# e-Figure 7. Distributional balance of covariates before and after propensity-score matching.


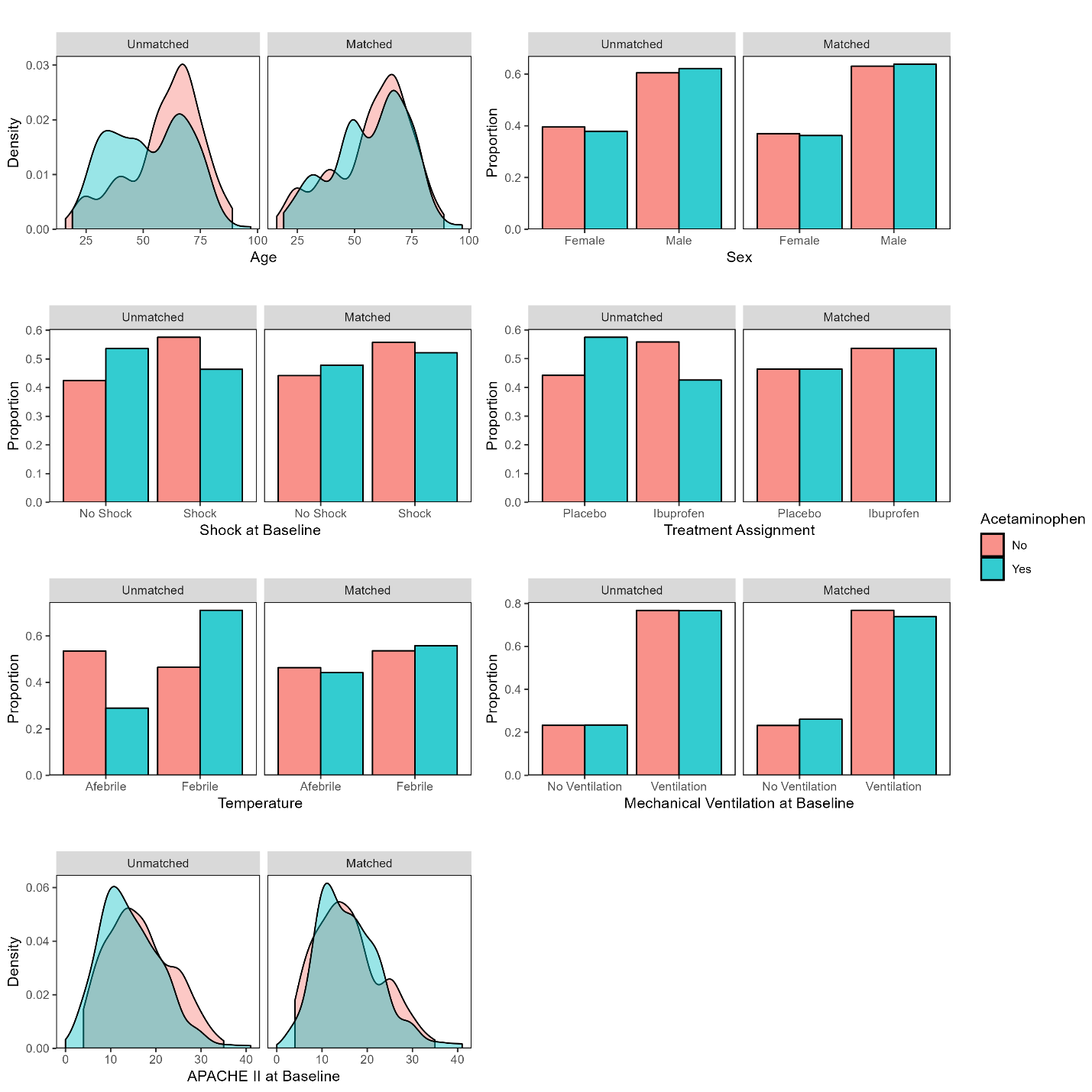


Left plot of each panel depicts the distributional balance of a variable in the unmatched cohort and the right plot of each panel of a variable depicts the distributional balance in the matched cohort. Density plots are used for continuous variables and bar plots are used for categorical variables. The plots show that after matching, acetaminophen has improved distributional balance overall among the covariates.

# e-Figure 8. Subgroup analyses of acetaminophen effect on ventilator free days across covariate subgroups in the Propensity Matched Cohort


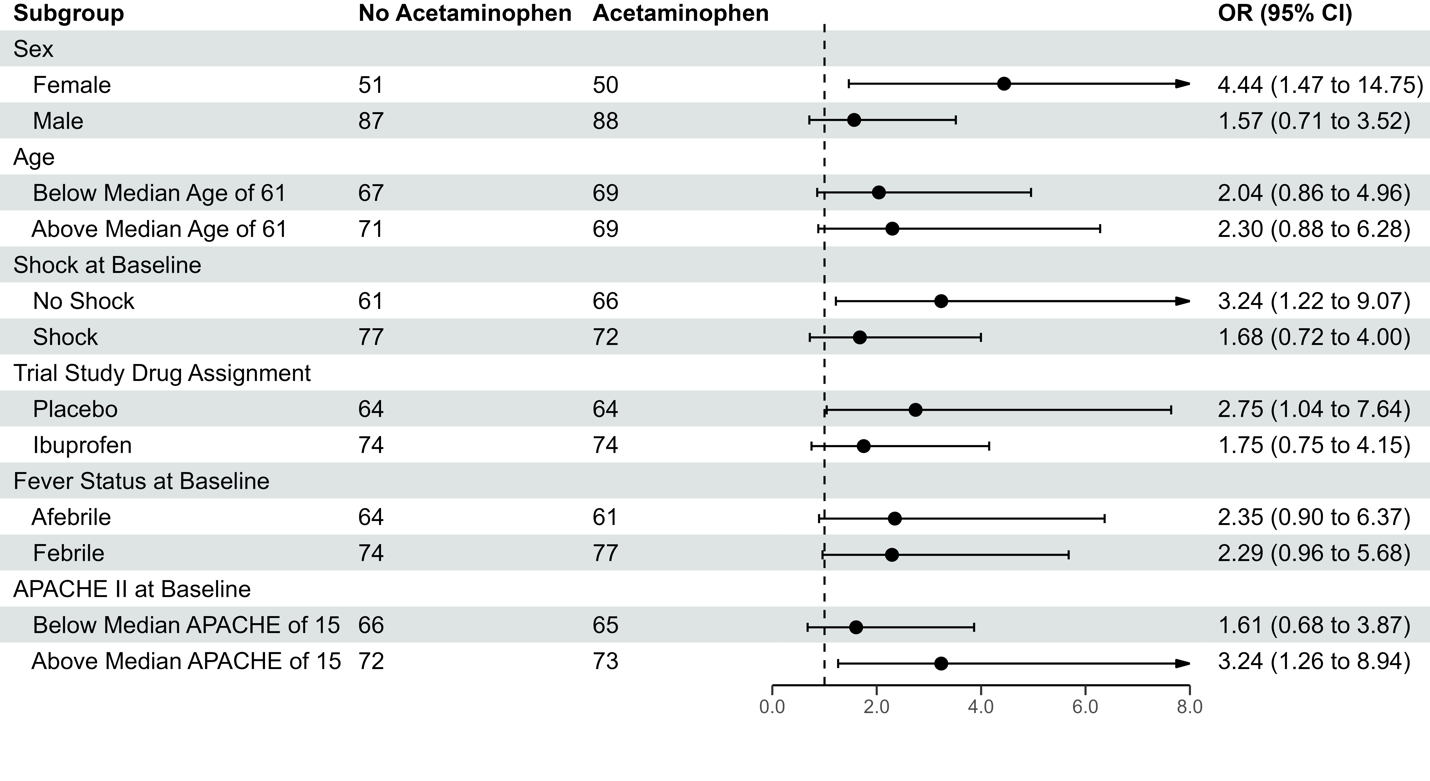


In the forest plots, the dots are point estimates, the bars are confidence intervals, and the vertical dotted line represents a odds ratio of one.

# e-Figure 9. Subgroup analyses of acetaminophen effect on renal failure across covariate subgroups in the Propensity Matched Cohort


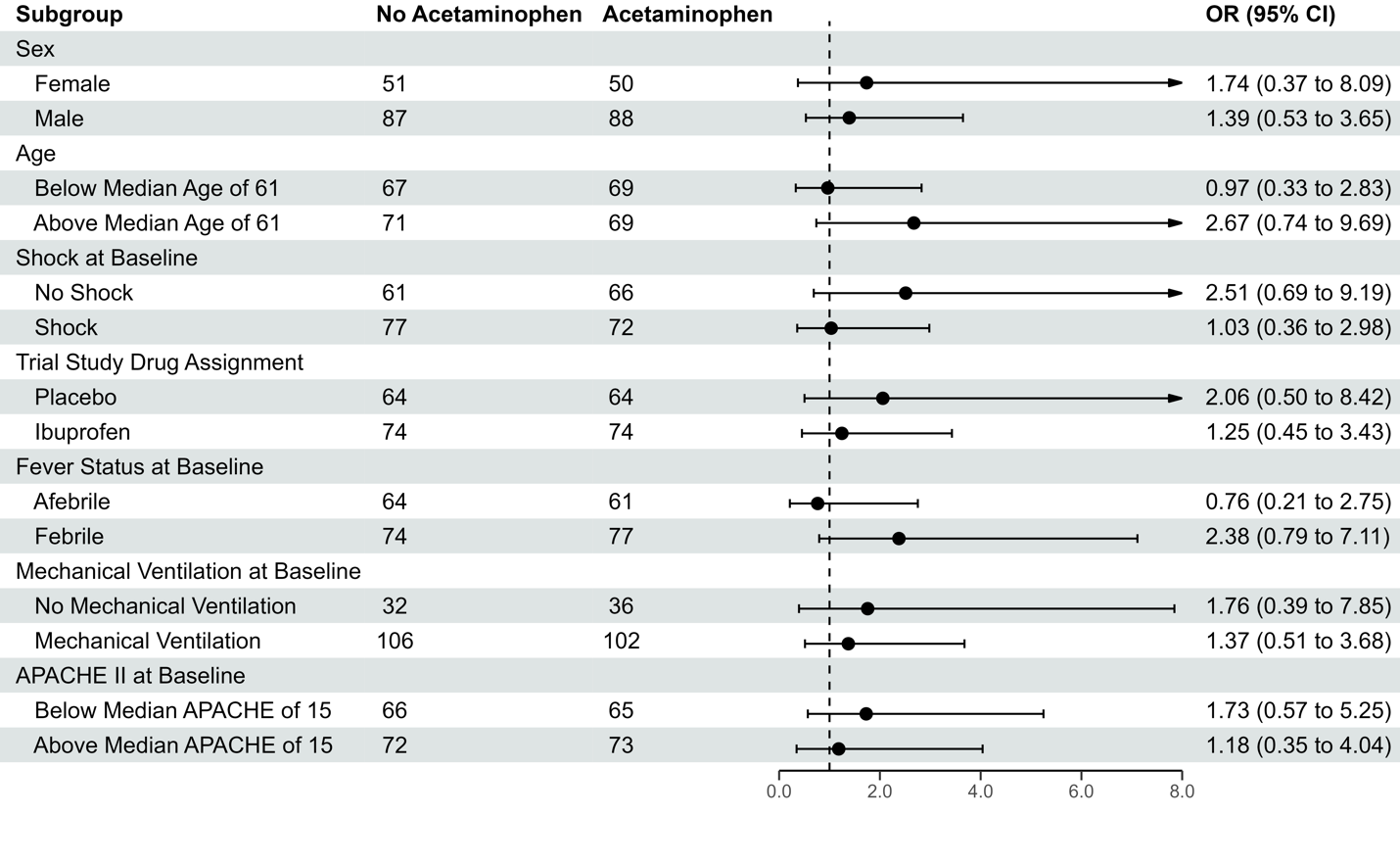


In the forest plots, the dots are point estimates, the bars are confidence intervals, and the vertical dotted line represents a odds ratio of one.

# e-Figure 10. (A) Kaplan-Meier Survival Curve for Stratified Propensity Matched Analysis of Acetaminophen use within the First Two Days and (B) Comparison of Ventilator-Free Days between Acetaminophen-Exposed and Acetaminophen-Unexposed Patients in the Stratified Propensity Matched Analysis in the First Two Days.


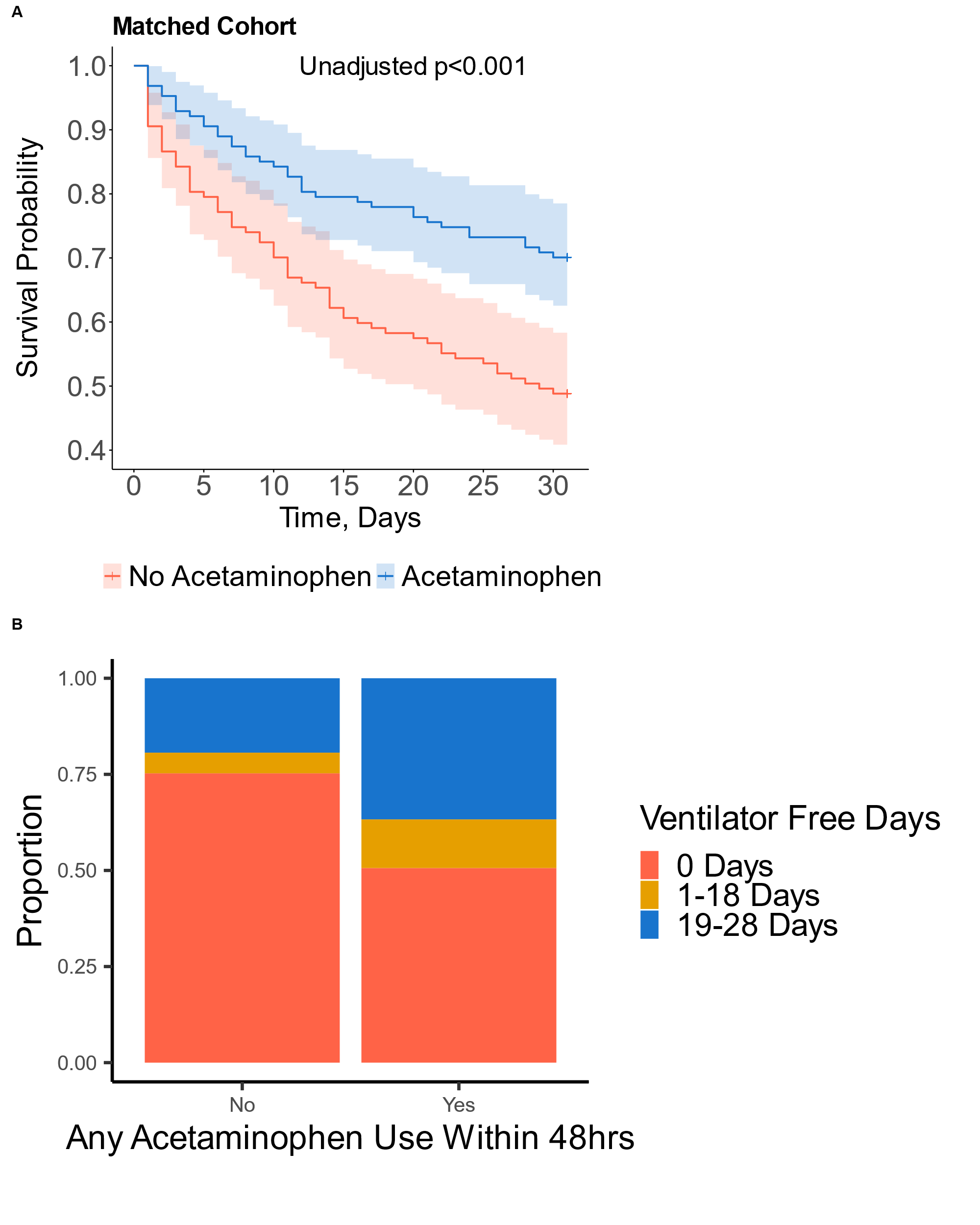


# e-Figure 11. (A) Kaplan-Meier Survival Curve for Propensity Matched Analysis of Acetaminophen use within the First Five Days and (B) Comparison of Ventilator-Free Days between Acetaminophen-Exposed and Acetaminophen-Unexposed Patients in the Propensity Matched Analysis in the First Five Days.


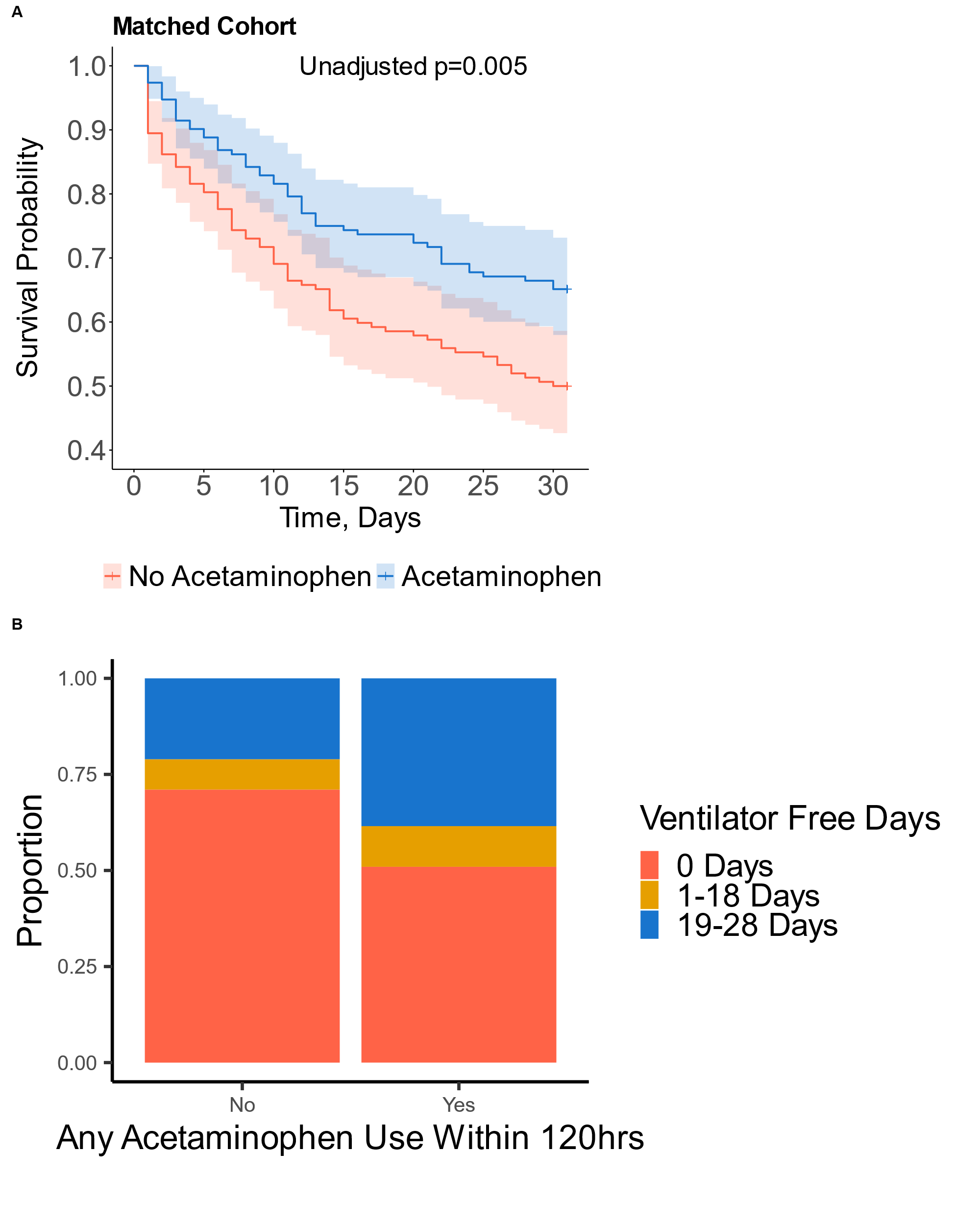

Supplement: 1 [file NIHMS2066139-supplement-1.docx]
